# miRNA therapeutics in precision oncology: a natural premium to nurture

**DOI:** 10.37349/etat.2022.00098

**Published:** 2022-08-31

**Authors:** Chakresh Kumar Jain, Poornima Srivastava, Amit Kumar Pandey, Nisha Singh, R Suresh Kumar

**Affiliations:** 1 Department of Biotechnology, Jaypee Institute of Information Technology, Noida 201307, India; 2 Amity Institute of Biotechnology, Amity University Haryana, Panchgaon, Manesar, Haryana 122413, India; 3 Department of Bioinformatics, Gujarat Biotechnology University, Gandhinagar, GIFT city 382355, India; 4 Molecular Genetics Lab, Molecular Biology Group, National Institute of Cancer Prevention and Research (ICMR), Noida 201307, India; Regina Elena Cancer Institute, Italy

**Keywords:** microRNAs, miRBase, miRTarBase, cancer, liposomes

## Abstract

The dynamic spectrum of microRNA (miRNA) has grown significantly over the years with its identification and exploration in cancer therapeutics and is currently identified as an important resource for innovative strategies due to its functional behavior for gene regulation and modulation of complex biological networks. The progression of cancer is the consequence of uncontrolled, nonsynchronous procedural faults in the biological system. Diversified and variable cellular response of cancerous cells has always raised challenges in effective cancer therapy. miRNAs, a class of non-coding RNAs (ncRNAs), are the natural genetic gift, responsible to preserve the homeostasis of cell to nurture. The unprecedented significance of endogenous miRNAs has exhibited promising therapeutic potential in cancer therapeutics. Currently, miRNA mimic miR-34, and an antimiR aimed against miR-122 has entered the clinical trials for cancer treatments. This review, highlights the recent breakthroughs, challenges, clinical trials, and advanced delivery vehicles in the administration of miRNA therapies for precision oncology.

## Introduction

**Figure 1.**
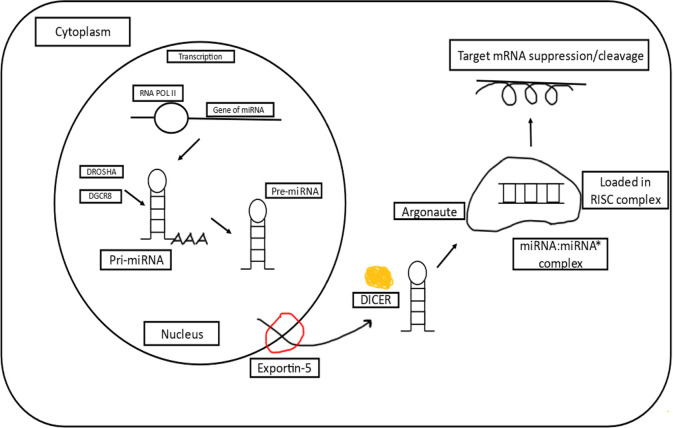
A basic overview of miRNA biogenesis [15]. RNA POL II: RNA polymerase II; DGCR8: DGCR8 microprocessor complex subunit

microRNA (miRNA) is known as the regulators of protein-coding genes that bind to the 3’ untranslated region of messenger RNA (mRNA) targets and exert their effect either by degrading the target mRNAs or the translational inhibition of those mRNAs [1, 2]. Mature miRNAs are the single-stranded RNAs (ssRNAs), also known as ssRNAs with ~20–25 nucleotides that depict an essential role in practically all physiological functions, involving the proliferation of cells, differentiation, apoptosis, and angiogenesis [3–6]. miRNA genes make up around 1% of the human genome and are evolutionarily conserved in practically all animals. Furthermore, post-transcriptionally modified miRNAs are thought to control more than a third of all the known genes coding for protein [7]. Following the discovery of evidence connecting carcinogenesis in human chronic lymphocytic leukemia (shown) to miRNAs [8, 9], the potential of miRNAs in the diagnosis, prognosis, and therapy in a wide range of cancers has sparked considerable interest [10, 11]. miRNAs have become intriguing tools and targets for novel therapeutic approaches thanks to new insights into their role in cancer. miRNA dysregulation has been linked to cancer in most cases, with miRNAs serving as tumor suppressors or oncogenes and mimics of miRNAs and compounds that target antimiRs have showed promise in preclinical development. A tumor suppressor miRNA mimic miR-34 has entered the first phase of clinical trials for cancer treatments, and also the antimiR which has aimed against miR-122, has come up to the second phase of clinical trials for treating hepatitis, these are among the miRNA-targeted treatments under development [12]. In April 2013, MRX34 the first cancer-targeted medicine designed via using miRNA which was a liposome-based mimic of miR-34, began with the first phase of clinical trials in patients with hepatocellular carcinoma (HCC), miRNA treatments are getting a lot of interest from academics and biotech businesses worldwide [13]. The majority of mature miRNA sequences are found inside the non-coding introns and exons, along with introns of precursor mRNA (pre-mRNA). The majority of miRNA genes are transcribed as large primary miRNAs (pri-miRNAs) by RNA polymerase II, which contain one or a few stem loop structures of about 70 nucleotides each. DROSHA cleaves pri-miRNAs into stem loop structures called precursor miRNAs (pre-miRNAs) in the nucleus. Exportin 5 transports pre-miRNAs to the cytoplasm, where DICER cleaves them close to the loop into structures called small double-stranded RNAs. The duplex of miRNA is then loaded onto the argonaute protein, which promotes the formation of a ribonucleoprotein complex also known as the RNA-induced silencing complex (RISC). Further, base pairing guides mature miRNAs to the 3’ end of their target mRNA, causing mRNA destabilization and translational repression as depicted in Figure 1 [14].

## Role of miRNAs in cancer

In various studies, miRNAs have been demonstrated to play a major role in cancer. Many of the miRNAs are highly expressed in cancerous cells and aid their progression [16]. Such as, miR-126 is known to be highly elevated in breast [17] and colorectal cancers [18]. Also, the genes associated with miRNA and the components of human cancer in miRNA machinery have been linked to tumorigenesis [19]. For example, DICER expression has been demonstrated to be downregulated in lung cancer [20]. The argonaute proteins, which are essential components of the RISC and control gene regulation via short interfering RNA (siRNA) and miRNA, have also been linked to cancer. Three human argonaute genes, argonaute RISC catalytic component 3 (AGO3), AGO1, and AGO4, are often deleted in Wilms tumors of the kidney and have even been linked to neuroectodermal tumors [21, 22]. AGO1 is highly expressed in the growing lung and kidney, and its expression is significantly higher in renal tumors lacking the suppressor gene of Wilms tumor, WT1 transcription factor (WT1) [21]. Some miRNAs, known as oncogenic miRNAs (OncomiRs), have varying degrees of expression in cancer and can influence cellular transition, metastasis, and carcinogenesis, by functioning as tumor suppressors or oncogenes. Although the multi-step aspect of tumorigenesis, the notion of “oncogenic addiction” suggests that targeting specific individual oncogenes might have therapeutic utility, and the idea of OncomiRs addiction had already been postulated but never proved. Overexpression of the miR-21 causes a precursor B-cell (pre-B-cell) lymphoma in one of the studies, suggesting that miR-21 is indeed a true oncogene. The tumors shrunk entirely in a few days after miR-21 was inactivated, mainly due to apoptosis. These findings show that tumors might be addicted to OncomiRs and lend support to ways to treat human malignancies by pharmacologically inactivating miRNAs like miR-21 [23]. Another study found that miR-125b can cause “OncomiR addiction” in the initial phase of cancerous progenitors by delayed differentiation and promoting a squamous cell carcinoma (SCC) cancer stem cell (CSC) transcriptional program in these cells [24]. Another study found that inhibiting miR-20a and miR-17-5p with antisense oligonucleotides (ASOs) might trigger death preferentially cancer cells of lung, overexpressing miR-17-92, indicating that a fraction of lung malignancies may be addicted to the production of these miRNAs [25]. Similarly, one more study found that a miR-155 feedback mechanism in Theileria transformed leukocytes is causing OncomiR addiction [26]. Moreover, miRNAs have been observed to be one of the keys to combat therapeutic resistance in breast cancer. Despite advancements in diagnosis and therapy, the incidences of cancer recurrence and drug resistance appear to be higher than ideal. Modifications in miRNAs have indeed been related to changes in essential cancer progression and development mechanisms. Various *in vitro *and *in vivo *researches have confirmed their role in breast cancer therapy resistance or sensitivity. Hence, the use of miRNA-based therapeutics to combat various types of cancer resistance is extremely promising [27].

## Therapeutic regulation of miRNAs in cancer

**Figure 2.**
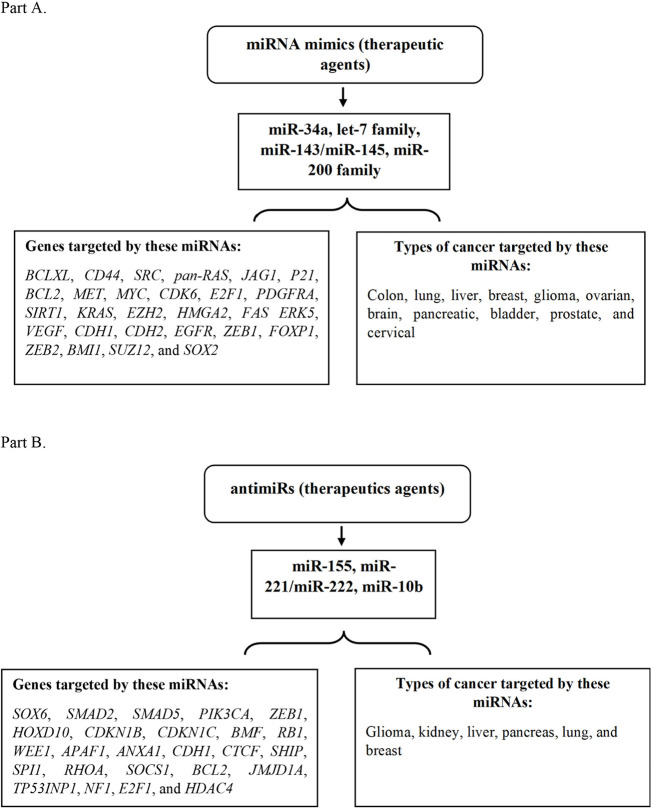
Tumor suppressive and Oncomeric miRNAs therapeutic as mimics and antimiRs. Part (A): depiction of some of the miRNA mimics as therapeutic agents targeting genes and various types of cancer. Part (B): illustration of a few of the miRNA antimiRs as therapeutic agents targeting the genes and different kinds of cancer. *KRAS*: *KRAS *proto-oncogene; *BCL2*: B-cell lymphoma 2

The potential to manipulate miRNA representation and function *in vivo *using mimics or antimiRs of miRNAs could lead to the development of novel cancer therapies [12]. We will discuss some of the strategies that are presently being tested in the lab replenishing tumor-suppressing miRNAs using their mimics or suppressing OncomiRs using their antimiRs. For instance, some of the miRNA therapeutics are being used in targeting cancer, and their clinical status [12] have been described in Figure 2 and Table 1.

**Table 1.** List of miRNAs based therapeutics in their clinical trials

**Therapeutics based on miRNAs and names of companies**	**Therapeutic agent**	**The system used for delivery**	**Disease targeted**	**Stage of clinical trial **	**Gov identifier of clinical trial**
Miravirsen (Santaris Pharma)	AntimiR-122	Antisense inhibitor with LNA modification	Chronic hepatitis C	Phase I is completed	NCT00688012, NCT00979927, NCT01646489
RG-101 (Regulus-therapeutics)	AntimiR-122	AntimiR conjugated with GalNac	Chronic hepatitis C	Phase II is completed	EudraCT numbers 2015-001535-21, 2015-004702-42
RG-125/AZD4076 (Regulus-therapeutics)	AntimiR-103/107	AntimiR conjugated with GalNac	Patient with type 2 diabetes	Phase I is completed	NCT02612662, NCT02826525
MRG-106 (miRagen-therapeutics)	AntimiR-155	Antisense inhibitor with LNA modification	Mycosis fungoides and cutaneous T cell lymphoma	Phase I is completed	NCT02580552
MRG-201 (miRagen-therapeutics)	miR-29 mimic	miRNA duplex with cholesterol conjugation	Scleroderma	Phase II is completed	NCT02603224
MesomiR-1 (EnGeneIC)	miR-16 mimic	Delivery vehicle of EnGeneIC	Mesothelioma and NSCLC	Phase I is completed	NCT02369198
MRX34 (miRNA therapeutics)	miR-34 mimic	Lipid nanoparticles	Multiple-solid tumors	Phase I is terminated	NCT01829971

EudraCT numbered trials are registered at EU Clinical Trials Register (clinicaltrialsregister.eu). Gov: government; LNA: locked nucleic acid; NSCLC: non-small cell lung cancer; GalNac: *N*-acetyl-galactosamine

### miR-34a

The most advanced miRNA therapies for cancer are miR-34 mimics encapsulated in lipid nanoparticles, which are now being investigated in the first phase of the clinical study (NCT01829971) in many hematological cancers. Many preclinical investigations have shown that miR-34 mimics have the potential to be an anticancer treatment. In mice models of the liver [28], prostate [29], and lung cancer [30], for example, the miR-34 mimic which was encapsulated in lipid nanoparticle exhibited assuring action. Systemic distribution of miR-34a in carriers of liposomes resulted in lower growth of the tumor, enhanced tumor cell death, and reduced CD44^+^ cell numbers in a model of orthotopic pancreatic cancer employing cells of MiaPaca2, depicting a reduction in metastatic cells [31]. In a prostate cancer mouse model, neutral-lipid emulsion (NLE)-based administration of miR-34a resulted in only a minor decrease in tumor development. However, due to a decrease in metastatic dissemination to the lung and other organs, a considerable improvement in survival periods was observed [29]. In an aggressive NSCLC *KRAS*; p53 tumor suppressor gene (*Trp53*) mouse model, therapy with a miR-34a mimic MRX34 which was coated in nanoparticles of lipid that are currently licensed for human clinical trials resulted in considerable tumor reduction [28]. Furthermore, in this model, a combined strategy employing the same lipid nanoparticle carrier to deliver let-7 and miR-34 resulted in a substantial decrease in nodules of tumor and a prolonged viability benefit. Also, *in vitro*, a combination of the epidermal growth factor receptor (EGFR) inhibitor erlotinib with the miR-34a and let-7 inhibitors exhibited coordinated effects in suppressing the development of cell lines of NSCLC [32].

### miR-200 family

miR-200c targets the interleukins, and delivery of the mimics of miR-200 family members utilizing the 1,2-dioleoyl-sn-glycero-3-phosphatidylcholine (DOPC) lipid nanoparticles resulted in fewer tumor nodules and distant metastasis in an orthotopic mice models of ovarian (miR-200a/b), basal-like breast cancer (miR-141), and lung (miR-200a/b) cancers [33].

### miR-26a

miR-26a levels were considerably shown lower in a wide range of RNA samples taken from individuals with HCC of *n *= 455 as compared to normal tissues [34]. Low levels of miR-26a were also linked to poor patient survival [35]. The specific targeting of mRNAs encoding for the cell cycle regulators cyclin D2 (CCND2) and cyclin E2 (CCNE2) by expression of adeno-associated virus mediated by miR-26a resulted in considerable tumor reduction in a mouse model of HCC [34].

### miR-506 and miR-520d-3p signaling

In two researches, DOPC liposomes involving miR-506 mimics which is a regulator of DNA damage response, and epithelial-mesenchymal transition (EMT) phenotype [36] or miR-520d-3p mimics which target the Ephrin type-A receptor 2 (EPHA2) and EPHB2 oncogenes [37] were delivered to orthotopic mouse models of ovarian cancer, suppression of ephrin signaling by miR-520d-3p resulted in remarkable tumor suppression and reduced expression in the respective mRNA targets *in vivo*.

### miR-15a/16-1

In MEG01 subcutaneous model of leukemia, the ectopic expression of clusters of miR-15a/16-1 via viral vectors resulted in a considerable decrease in tumor volume and growth [38]. Furthermore, administration of miR-16-1 via a nano-cell delivery system which is an EGFR-targeted Delivery Vehicle (EDV) of EnGeneIC in NSCLC xenograft mice models and malignant pleural mesothelioma, led to the targeted delivery in tumor and considerable tumor repression [39].

### miR-10b

Anticancer techniques on the basis of OncomiRs suppression by utilizing antimiRs based on antagomirs, LNAs, and ASOs have been examined in several preclinical investigations [12]. ASOs were utilized to successfully inhibit miR-10b in an orthotopic breast cancer model in an early evaluation of the therapeutic value of antimiRs [40]. Metastasis was reduced because of the expression of the homeobox D10 (HOXD10) anti-metastatic gene being rescued by this antimiR. However, the authors found no reduction in original tumor development, indicating that the first combinations of chemotherapy and tumor reduction surgery are required. It would be fascinating to observe if miR-10b inhibitors have an effect on long-term survival. Delivery of an antagomir against miR-10b utilizing a polyethyleneimine (PEI) delivery technology which was a *in vivo*-jet PEI, resulted in a substantial decrease in the tumor development in an orthotopic glioblastoma mouse model [41]. Given that HOXD10 is not considered to play a role in the original tumor’s development, this finding requires more investigation. It also shows that miR-10b has significance other than downregulating HOXD10.

### miR-221

In HCC, miR-221 is one of the most dramatically elevated miRNAs, downregulating important tumor suppressors such as p27KIP1, phosphatase and tensin homolog (PTEN), and tissue inhibitor of metalloproteinase 3 (TIMP3) [42, 43]. A cholesterol-modified version of antimiR-221, given intravenously to HCC xenografts, was found to have considerable effectiveness in downregulating miR-221 and raising the levels of its mRNA targets [44]. Tumors were reduced in antimiR-221-treated animals, and they lived substantially more than the control mice. The utilization of this cholesterol-modified antimiR for future research is currently limited due to a lack of comprehensive toxicity data.

### miR-155

It was demonstrated via the model of a mouse miR-155-induced with lymphoma in which the expression of miR-155 was controlled by doxycycline (miR-155^LSLtTA^), that removing doxycycline caused miR-155 expression to shut down and tumor reduction beyond detectable limits [45]. The introduction of antimiR-155 nanoparticles packed in nanoparticles of polylactic-coglycolic acid resulted in a lower burden of tumor in the same mouse model, indicating that miR-155 inhibition may have therapeutic potential. pH low insertion peptide (pHLIP)-antimiR-155, a pH-sensitive antimiR-155 conjugate, was recently investigated in this model [46]. A short peptide called pHLIP creates a helix of transmembrane within acidic conditions [47]. Because the tumor milieu is acidic, a pHLIP and antimiR-155 combination boosted antimiR distribution to cancer cells. Mice that were given pHLIP–antimiR-155 had a considerable decrease in tumor burden, which resulted in a longer survival time. There was no notable toxicity, indicating that clinical translation of this method is possible [46].

### miR-630

miR-630 is an OncomiR that is elevated in response to hypoxia in a tumor-specific environment. In an ovarian cancer orthotopic model, it was observed that via using the antimiR that was against the miR-630 through the DOPC delivery system there was a substantial decrease in the metastasis and the growth of the tumor [48].

## miRNA-focused computational resources

At present, there are various databases that have been developed which have the miRNA registries, miRNA targets, their disease association, and differential expression, and also some all-in-one resources [49].

### Repositories for miRNA discovery

For the identification of miRNAs in the laboratory carrying out the experiments like cloning, microarray, and *in-situ *hybridization methods are all very much time-consuming and also expensive methods. However, in recent years, next generation sequencing (NGS) has made miRNA detection simple and less costly. As a result, hundreds of miRNA sequences for animals, plants, and viruses have been identified. Many techniques have been designed which utilize the high throughput sequencing data to find out new miRNAs. These methods are based upon a sequence-structures factor that is unique to miRNAs, such as the composition of the sequence, the conservation across the species, hairpin loop location and number, the number of base pairs and bulges, and all the structural parameters such as the Gibbs free energy and secondary structures [49]. As a result, a miRNA Registry was created to meet the storage needs of these miRNA sequences. Over 24,000 miRNA genes are represented in miRBase [50, 51], with over 30,000 mature miRNAs and extensive annotations from over 200 species [52]. There are more than 1,800 pre-miRNAs in humans and more than 2,500 mature miRNAs that are included in the current edition. Plant microRNA database (PMRD) [53], a repository containing miRNAs from plants, is an example of a species-specific database that provides more extensive coverage of experimentally confirmed miRNA sequences. Similarly, viral miRNA (VIRmiRNA) is the first dedicated resource to archive experimentally proven virus-encoded miRNAs, their targets, and antiviral miRNAs in three sub-databases. Epimir is a broad resource of common regulations between miRNAs and epigenetic alterations [54], and AVIRmiR, is a sub-database of VIRmiRNA that encompasses host’s encoding miRNAs that are reported to have anti-viral effects [55], these are the two examples of repository which holds miRNAs according to particular conditions or experimental principles used to extract them. Some databases provide miRNA annotations as well as visualization tools, such as miRviewer, which is a Web service that visualizes miRNAs via miRBase and also their homologs which are identified by utilizing miRNAminer [56]. Moreover, the miRBase tracker, from the name itself, explains that it keeps the researchers up to date on changes in miRNA annotation and makes it easier to annotate [57]. MirGeneDB, on the other hand, employs a historical hierarchy to provide comprehensive annotations of miRNA:miRNA^*^ duplex [58]. Others, such as miROrtho, which is an online program that gives predictions of pre-miRNA from genomes of animals in conjunction with their orthology [59], and also focuses on both the evolutionary and storage aspects of miRNA. CoGemiR [60] is an online database that proposes conservation across the evolution. Furthermore, mESAdb is an online database that contains expression data of miRNAs from a variety of species [61]. As a result of advancements in NGS-based approaches, several other depositories for archiving predicted miRNAs and multiple other aspects of NGS-based information, such as YM500v2—which is an archive for predicting and quantifying miRNA and related isomiRs from short-RNA sequencing data sources, have been established. In its current edition [62], cancer miRNAome research is also included. miRNEST is a database that contains experimentally predicted miRNAs from a variety of animals [63]. MirPub, a comprehensive database with a search capability that provides researchers with papers relevant to specific miRNAs [64], also, in the field of miRNA, is one of the literature searches enhancing depositories.

### Differential expression of miRNAs

miRNAs are known to be gene expression regulators, and their altered expression has been linked to a variety of illnesses. Real-time polymerase chain reaction (PCR), southern blotting, and NGS-based methods have recently been utilized to characterize the differential expression of miRNAs using several miRNA profiling methodologies. Because of their precision and speed, NGS-based approaches have grown popular. As a result, methods that reveal differentially expressed miRNAs will be tremendously valuable in discovering biomarkers for a wide range of diseases. Scientists have created a unique category of resources for storing and analyzing DEmiRs [49]. MirEX 2.0 database [65] and human miRNA expression database (HMED) [66] are two computational tools that include miRNA expression profiles. MirEX and HMED are two powerful tools for analyzing miRNA expression data. DEmiRs may also be found in several databases, such as bloodmiRs, which include miRNAs that are cell-specific in the peripheral blood of humans [67]. Likewise, ExcellmiRDB [68] has been the first database specialized in DEmiRs in biofluids with vetted data. miRandola, on the other hand, gives extensive information on numerous extracellular circulating miRNAs [69]. During every disease, environmental variables, as well as miRNAs, play an important influence. A database called miREnvironment was created to hold empirically verified data revealing correlations between environmental factors, miRNAs, and their associated phenotypes [70]. Some sites are dedicated to storing differentially expressed miRNAs from the tissues of the plant; for example, PmiRExAt is a repository in the early stages of development that has a complete view of plant miRNA expression profiles across a number of tissues. They also looked at the expression patterns of miRNAs using data from publicly available short RNA datasets [71]. HPVbase [72] summarizes the expression patterns of differentially controlled miRNAs associated with HPV infection in great detail.

### Bioinformatics databases and tools enclosing targets of miRNAs

The finding of a vast amount of miRNAs expressed in different species raised a critical question concerning their function. In order to answer this question, cloning and computational techniques were developed to find their targets. TarBase [73] is the most comprehensive library of experimentally confirmed miRNA targets from a variety of species. miRTarBase [74, 75] is a database that tracks the target interactions of miRNAs. miRGate is a database that is manually maintained and it examines miRNA and its gene targets, and also isoforms, from humans, mice, and rats 3’ (untranslated region) UTR regions [76]. miRNA targets that are conserved in five vertebrates are provided by TargetScanS. Further, the plant non-coding RNA database (PNRD) is a specialized tool for miR-targets and other non-coding RNAs (ncRNAs) in plant species. It now has over 25,000 entries of around 10 different types of ncRNAs from over 150 plant species [77]. Likewise, VIRmirTar is a subdivision repository of the VIRmiRNA coordinated resource that contains a wealth of knowledge on the cellular and viral targets of miRNAs expressed by viruses [55]. Both experimental and computationally anticipated data may be found in certain well-known archives. miRecords is a comprehensive repository of miRNA-target interactions. It is made up of two sub-databases. The former includes carefully selected, empirically confirmed miRNA targets gleaned from thorough literature searches, whereas the other includes targets that are computationally predicted. These forecasts are governed by eleven well-known prediction systems [78]. Moreover, miRWalk is a free, all-encompassing resource that provides empirically validated and projected miRNAtarget interaction pairings [79]. Seven commonly known miRNA target gene prediction methods have been added to miRSystem: PicTar, DIANA, TargetScan, PITA, miRBridge, RNA22, and miRanda [80]. There exists computational prediction of miRNA-binding regions in the sequences of the promoter of ideal sequences; for instance, microPIR2 is a repository of computationally determined miRNA target sites in human and mouse genome promoter sequences. It also includes investigative information on expected targets. [81]. Similar to miRBase [82], ViTa volunteers scientifically identified host miRNA targets.

miRTar is a repository to observe miRNA-target relations [83], while DIANA microT-coding sequence (CDS) which is also an online database for predicting miRNA-target relations [84]. Depositories containing projected miR-target interactions are typically of two different types: those that carry information from numerous species and/or those that are specialized to a single species. MicroCosm contains miRNA targets that have been computationally predicted for a variety of taxa. More than of about 2 million putative gene targets are controlled via more than 6,500 miRNAs, according to miRDB [85, 86]. The EMBL’s miRNA—Target Gene Prediction repository contains information on miRNA-targets gene prediction for the fruit flies. Some of the computational data sources, such as multiMiR, a catalog-cum-miRNA-target R program, allow target prediction by combining accessible techniques with other connected functionalities. This portal provides access to over 50 million human and mouse records from a variety of databases [87]. Similarly, the CSmiRTar database, which stands for the condition-specific miRNA target database, makes predictions that are based on various situations [88]. Single nucleotide polymorphism in the miRNA-binding sites of the genes has been shown to affect the miRNA target repertoire. miRSNP, miRdSNP, and PolymiRTS were created to cope with the existing biological data in these factors. miRSNP is a collection of human miRNAs predicted single nucleotide polymorphisms (SNPs). Likewise, miRdSNP provides a complete database of single nucleotide polymorphism on human genes’ 3’ UTRs [89]. PolymiRTS is a database-as-a-service that may be used to investigate the impact of SNPs in miRNA seed sequences. Users can investigate the relationships between SNPs and gene expression features, pathways, and disease phenotypes [90, 91].

### Bioinformatics tools for miRNA disease associations

Additionally, archives containing miRNAs organized by illness or experimental systems used to elicit them are given. HMED, for example, is a database that includes human miRNA expressions [66]. EpimiR is a comprehensive database of epigenetic alterations and miRNA associations. CoGemiR is another online server that tracks the development of miRNAs in diverse animal species. OncomiRD, miRCancer, and dbDEMC are catalogues of differentially expressed miRNAs from different kinds of malignancies [92, 93]. Further, PhenomiR is an online resource that provides data regarding DEmiRs and their roles in numerous diseases and biological processes [94]. PMTED was created to assess miRNA expression patterns and also their targets which are from the published plant micro-array research [95]. Furthermore, miRStress is an online database that contains miRNAs linked to stress [96]. The Dietary MiRNA Database (DMD) contains reported miRNA from human meals [97]. PASmiR was created with the goal of creating software for collecting, standardizing, and querying data on miRNA-stress regulations in the plant. Likewise, EpimiRBase is a handy database that contains data on miRNAs that have been linked to epilepsy [98]. Similarly, antiviral miRNAs during viral infections are covered by AVIRmiR, a VIRmiRNA type of database, some of the resources are described in Table 2 [55].

**Table 2.** A whole repertoire of miRNA resources

**Databases**	**Organism**	**Specification**	**Weblink**	**References**
miRNAMap	Human, rat, fly, worm, chicken, mouse	miRNAs and target genes experimentally verified	http://mirnamap.mbc.nctu.edu.tw/	[99, 100]
microRNA.org	Human, mouse	Target predictions of miRNA	http://www.microrna.org	[101]
PMTED	Plants	Microarray studies have revealed expression patterns of miRNA targets	http://pmted.agrinome.org	[95]
MtiBase	Human, mouse	miRNA-binding sites located at 5’ UTR/CDS	http://mtibase.sysu.edu.cn	[102]
miRGator	Human	Expression profiles, miRNA diversity, target relationship	http://mirgator.kobic.re.kr	[103, 104]
SomamiR DB 2.0	Human	Somatic-mutations in miRNAs and their target-site in cancer	http://compbio.uthsc.edu/SomamiR	[105]
PhenomiR	Human	Expression of miRNAs differentially regulated in diseases	http://mips.helmholtz-muenchen.de/phenomir	[106]
DIANA miRGen v3.0	Human, rat, mouse	Cell line specific genes transcription-start-sites (TSSs) of miRNA	http://www.microrna.gr/mirgen	[107]
PASmiR	Plants	A Web-accessible and literature-curated database	http://hi.ustc.edu.cn:8080/PASmiR	[108]
DIANA TarBase	Animals, plants, virus	Experimentally validated miRNA and gene interactions	http://www.microrna.gr/tarbase	[109]
miR2Disease	Human	Deregulation of miRNAs in various human diseases	http://www.mir2disease.org/	[110]
mimiRNA	Human	Expressions-profile data of miRNA over various cell lines and tissues	http://mimirna.centenary.org.au	[111]
StarBase	Human, mouse	miRNAs, competing endogenous RNAs (ceRNAs), pan-cancer and interactions network of long ncRNAs and mRNAs from a large-scale CLIP-sequence data	http://starbase.sysu.edu.cn/	[112, 113]

## Disruption of miRNA in cancer: general processes

Since their discovery, abnormal miRNA expressions have been related to a variety of clinical conditions, involving oncology. In fact, more than half of all miRNAs have been found in cancer-related genomic regions [114]. The profiling of expression and following characterizations of miRNAs associated with cancer added to the evidence that miRNAs are important in cancer research. It has been discovered that miRNA expression rises mostly during differentiation. As a result, because cancer is frequently associated with overall dedifferentiation or failure of the differentiation process, it was expected that miRNA expressions would be reduced widely. In chronic lymphoblastic leukemia, miR-16 and miR-15, which are commonly omitted in these tumors, were revealed to be negatively regulating the anti-apoptotic gene of BCL2 [9, 115]. This was the first evidence of miRNAs playing a causative role in cancer. In the context of cancer, structural changes affecting miRNAs are a typical route for miRNA disruption. miRNA dysregulation is caused by DNA methylation of promoters, activation of critical oncogenic transcriptional factors, and anomalies in the miRNA biogenesis process. Transcriptional repression by oncogenic transcription factors is frequently responsible for global miRNA repression. MYC proto-oncogene (MYC), which has been demonstrated to suppress a variety of miRNAs, involving miR-34 and let-7 family, which gather to induce proliferation [116], is one noteworthy example of this regulation. Because the let-7 miRNA family has been demonstrated to be tumor suppressive, it is commonly downregulated. Additionally, carcinogenic miRNAs have been identified, like miR-17-92 clusters, that have been demonstrated to enhance cell proliferation, decrease apoptosis (cell death), and induce the angiogenesis process [117]. Copy number variations and cytosine-phosphate-guanine (CpG)-methylation had been linked to the cancer-related miRNAs [118]. Also, a feedback loop between the OncomiR miR-155 and programmed cell death 4 (Pdcd4) with the activation of protein 1 modulates the miR-155 expression and tumorigenesis in tongue cancer [119]. The exact processes driving miRNA dysregulation in cancer, however, are unknown [120].

## Challenges

**Figure 3.**
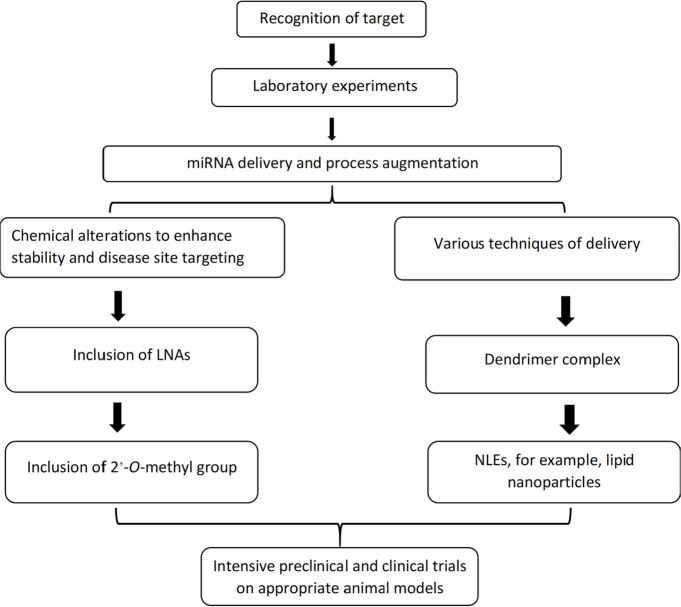
The major stages in the production of miRNA therapeutics are described. The initial step for the preparation of miRNA therapeutic is the proper identification of candidates of miRNA by *in vitro *experiments. The generation of chemical manipulations and mechanisms of delivery for miRNA antimiRs and mimics for the *in vivo* modeling is the next significant challenge. Chemical changes, such as the insertion of a 2-*O*-methyl group or LNAs, can dramatically improve stability. A lipid nanoparticle, like NLEs, and a dendrimer complex containing target moieties are two typical delivery strategies. After clearing these challenges, short RNA therapy candidates must go through extensive disease-specific *in vivo *experiments in animal models. To minimize early clinical trial failures, a careful review of toxicity studies and target engagement is essential [12]

Finding effective miRNA therapeutics for cancer is a challenging task. As RNA oligonucleotides have properties that make medication design and effectiveness more difficult. The following qualities are challenging: (i) nuclease degradation when added to biological systems [121, 122]; (ii) a lack of penetration into the cell membrane [123]; (iii) entrapment in the endosome [124]; (iv) a low affinity for complementary sequences in binding [125]; (v) ineffective distribution to target tissues [124]; (vi) activation of innate immune responses; (vii) off-target and undesired toxicities [126]. These issues are being addressed in a variety of ways since miRNA delivery represents a potentially unique treatment technique. For example, we show how chemical modifications to oligonucleotides can prevent naked miRNAs from being degraded by nucleases, whereas the hydrophilic properties, high molecular weight, and negative charges could prevent nucleic acids from permeating the cell membrane could be addressed with various delivery techniques as depicted in Figure 3 [127].

### In the bloodstream, unmodified miRNAs are rapidly eliminated and degraded

Maintaining stability and uniformity of miRNAs in circulations is a difficulty in miRNA therapies. In seconds, nucleases in the blood, such as serum RNase A-type nucleases, degrade naked miRNAs with an unaltered 2’-OH in the ribose moiety [128]. Furthermore, naked miRNAs are promptly excreted by the kidneys, which results in a shorter half-life in the systemic circulation. The chemically designed miRNA alterations on the phosphodiester-backbone and on the 2 of the ribose that prevents the miRNA from degrading and boosting its long-term efficacy are the solution to this problem [128, 129]. Phosphodiester links, ribose-backbone, 2’-*O*-Methyl, 2’-*O*-(2-Methoxyethyl), 2-Fluoro, and 2-locked-nucleic acid are some of the chemical changes that have been discovered. These changes enhance the stability of oligonucleotides while simultaneously increasing the binding-affinity towards the target and assisting loading it into the miRNA-induced silencing complexes (miRISCs), of which both boost miRNA function.

### *In vivo *and *in vitro *penetration of miRNA is limited

Hundreds of miRNAs have been discovered to have a role in the genesis and progression of illness, including cancer. Despite significant advancements in miRNA delivery, the primary difficulty of miRNA therapies is effectively delivering it to the targeted tissue with an efficient payload penetration onto a particular spot [130]. Poor blood perfusion is caused by the leaky nature of tumor blood vessels in cancer, which lowers the transport effectiveness of bare miRNAs.

#### Different delivery vehicles have been created to address this limitation:

(i) Methods based on nanocarriers (liposomes): this takes advantage of leaky tumor arteries in which the enhanced permeability and retention (EPR) effect is used that is caused by the leakiness of tumor-associated neo-vasculature, which allows nanoparticles with appropriate size distribution to concentrate in the tumor microenvironment compared to healthy tissues [131].

(ii) In a study, *in vivo *delivery method using polymeric nanoparticles to deliver miRNA [miRNA polymeric nanoparticles (miNPs)] for its localized administration within a shear-thinning injectable hydrogel to treat myocardial infarction (MI)-related heart failure has been demonstrated [132, 133].

(iii) Similarly, techniques based on conjugation, in which a lipid, sugar, or peptide is covalently attached to the 3rd end of the aptamers, or passenger strand, or antibody conjugation for the tissue selectivity [134, 135].

(iv) Exosomes [136], and viral vectors [132] are two of the most common types of delivery techniques. In one of the types of research, the dynamic shedding of miR-22-loaded exosomes from the cytosol to the extracellular environment was observed for the first time [137]. In many of the studies, viral vector has also been used for *in vivo *delivery. Viral vectors cover a wide range of applications, including delivery vehicles designed for both transient and long-term expression. Adenoviruses are the most often used viral vectors. A large number of clinical experiments using viral vectors have been done or are now underway [138]. Nanoparticles have been utilized to transport vectors expressing miRNAs in some circumstances. Electrostatic interactions coupled the plasmid vector encoding the miRNA to a cationic polymer, which was subsequently loaded on nuclear pores [139]. Some of the other solid lipid nanoparticles (SLNs), liposomes, and nanostructured lipid carriers (NLCs) are also examples of lipid-based nanoparticles [140]. Because of their biocompatibility and biodegradability, both the polymers and liposomes are sub-types of nanoparticles that are highly effective in overcoming delivery problems. Furthermore, miRNA and other single or double-stranded oligonucleotide are charged negatively, resulting in poor tissue permeability. Now, the current delivery method works to create nanoparticles with variable sizes and characteristics owing to various microenvironments, situations, or time series. Nanoparticles are made up of components that allow for the regulated release and effective diffusion of therapeutic payloads into sick tissues. In certain therapeutic experiments, miRNA administration using the nano-carriers for the down-regulated miRNA or suppression of overly expressed miRNA showed a satisfactory result in mitigating sick conditions [141]. When compared to naked miRNA, nanocarriers improve miRNA stability and transfection effectiveness into cells by a factor of ten.

### Escape of endosome is difficult

miRNA trafficking initiates in the primary compartment of the endosome, whether the miRNAs are supplied by nanoparticles, cell-type specific, or cationic lipid delivery vehicles. The contents of the early endosomes are then transferred to the late endosomes. The environment of late endosomal vesicles is acidic, i.e., around pH 5 to 6. The contents of the endosomes are subsequently transported to lysosomes, which are acidified with a pH of 4.5 and contain numerous nucleases which promote the breakdown of oligonucleotides-miRNA mimic. miRNA mimics should escape into the cytoplasm from the endosome, where they may interact with the RNA-interference machinery, to avoid lysosomal destruction. The endosomal uptake mechanism is recognized to represent a rate-limiting barrier in miRNA distribution. Endosomal vesicles capture bioactive compounds, which are then destroyed in the lysosomal compartment, requiring the development of efficient ways to enable endosomal escape and increase cytosolic bioavailability [142]. The endosomal release can be aided by a variety of methods: (1) lipoplexes with a pH sensitivity [124]; (2) polyplexes with a pH sensitivity [143]; (3) molecules that are photosensitive [144]; and (4) also with the use of cationic nanoparticles [145].

### There are various obstacles to extrahepatic-delivery

The well-known trivalent GalNAc compound binds with the receptor of asialo-glycoprotein on hepatocytes with great specificity and affinity, which results in targeted delivery of oligonucleotides and the gene-silencing in the hepatocytes. Further, the platform of GalNAc performance illustrates that efficient delivery of tissue of the therapeutic oligonucleotide is the cornerstone of each clinical trial. However, lipids in conjugation with miRNAs have been reported to improve the delivery of miRNA [146]. The liver accumulates the bulk of lipid conjugated miRNAs examined, such as cholesterol conjugated miRNAs (60–80%). Extrahepatic tissues, on the other hand, show accumulation and productive silencing of cholesterol-modified miRNAs. Furthermore, local injections of siRNA which are cholesterol-modified result in functional gene-silencing in the skin, brain, and vaginal canal [146]. Extra-hepatic tissue targeting of miRNAs remains a challenge, restricting the application of miRNA-based therapeutics. Despite the fact that polymer nanoparticles and lipids may be designed for effective endosomal escape and cellular absorption, they nonetheless aggregate in the spleen, liver, and kidney. Extra hepatic scaffolds, such as peptides, lipids, and antibodies that are coupled to particles, or aptamers, are used to overcome this difficulty, resulting in targeting by recognition of particular receptors [134, 147].

### Unwanted on-target effects and off-target effects

One of the primary challenges with miRNA treatment is the off-target effects of the miRNAs after they are transported in the cytoplasm and are released from the endosomes. miRNAs are made to particularly target certain pathways via faulty hybridization with the 3’ UTRs, but they might silence the other genes unintentionally. The off-target gene-silencing could result in toxicity and a reduction in the therapeutic efficacy. Besides, the potential of a single miRNA that could target several mRNAs warrants critical consideration since it suggests the risk of unforeseeable side effects. Moreover, even if a single miRNA is targeted adequately, undesired on-target effects may occur [148]. To solve such a barrier, one of the strategies is the utilization of modest dosages of coupled miRNAs that influence the expressions of the same targeted genes synergistically [149]. The co-transfections of the miR-15a/16 and miR-34a, for example, resulted in enhanced cell-cycle arrest in the NSCLC because of the fact that miR-15a and miR-16 selectively downregulate the CCNE1 and the CCND3, respectively. Such kind of gene-regulation has a complimentary impact towards the miR-34-mediated cell-cycle regulations [150].

### miRNAs have the ability to trigger the immune system

The host system may see double-stranded RNAs as pathogens, and the innate immune system may identify those and gets activated. Such as, like other forms of nucleic acids, systemic miRNA administration could trigger the innate immune system, resulting in undesirable and major toxic side effects. Through Toll-like receptors (TLRs), systemic injection of miRNA duplexes can activate the release of inflammatory cytokines and type I interferons (IFNs). Single or double-stranded RNAs could activate TLRs3, 7, and 8 to promote adaptive and innate immune responses. TLRs detect double-stranded RNA molecules in the cellular endosomes and lysosomes, triggering the IFNs pathway as well as cytokines synthesis [151]. Scientists have been currently researching immunological responses to miRNAs, but it appears that the chemical alterations also diminish immune system recognition [122]. The reaction of the immune system towards the delivery vehicles which are highly positively charged, which could also be toxic and might activate the immune system is also of great importance. Interactions with the immune systems are unavoidable when nanoparticles enter into the body. Furthermore, the surface modifications, size, hydrophobicity, and shape of the nanoparticles all have an influence on their interactions with the immune system [152]. As a result, both the miRNAs and the delivery system must be investigated. Reduced dose and hence presumably reduced immune responses will be possible if the carrier is targeted to certain organs [153].

## Future prospective

Over 2,000 miRNAs have already been discovered as being demonstrated in humans, and are thought to target and/or modulate the expressions of about 60% of genes of humans. Because abnormal miRNA expression is linked to a variety of disorders, including cancer, miRNA expression profiling is critical for understanding the etiology of practical cancers of all kinds. Furthermore, in contrast to widespread perception, miRNAs have recently been shown to have the ability to interact directly with one another. For example, miR-107 suppresses the expressions of let-7a, which is a tumor-suppressor, resulting in an increase in carcinogenic activity. Elevated levels of miR-107 were shown in advanced breast cancer cell lines, along with reduced levels of let-7a [154]. Similarly, the miR-15/16 cluster encodes miRNAs that are observed to act as tumor suppressors, and the *in vitro *and *in vivo *expression of such miRNAs decreases cell proliferation, induces cancer cell death, and lowers tumorigenicity. Several oncogenes, such as myeloid cell leukemia 1 (MCL1), BCL2, Wnt family member 3A (WNT3A), and CCND1 are targeted by miR-15a and miR-16-1 [155], at the posttranscriptional level in the nucleus, of mouse miRNA-709 has been observed to directly regulate the biogenesis of miR-15a/16-1 [156]. As a result, one of the most critical concerns in revealing the pathophysiology of cancer will be interpreting the functions of the direct correlation between the miRNAs. Because of their increased expression in tumor tissues and high stability in human bodily fluids, miRNAs have been recommended as biomarkers for the early identification and diagnosis of cancer. miRNAs are one of the most important hypothesized possibilities as recently existing diagnostic techniques are insufficiently specific and sensitive. Furthermore, despite the fact that miRNAs have a broad spectrum of targets because of the partial homology, their employment as a therapeutic application in cancer is unavoidable. To design effective and practical medicines, it is necessary to understand their function of how they work, their genuine target sites, and accurate expressions knowledge. As a result, there is a pressing need to learn more about the role of miRNAs in cellular function, as well as their oncogenic and tumor suppressive capabilities, by delving further into new discoveries about their expressions, activities, and functions in normal and diseased conditions [157].

## Conclusions

In this review, we highlighted the recent therapeutical advances, and clinical trials in the area of miRNA, with a focus on impending computational biology difficulties for finding new miRNA and targets. We have also explored and detailed the several computational databases and tools used in the field of miRNA study in order to assist the scientists in selecting the best databases for their scientific investigations, as well as instructions on how to use each dataset. Moreover, despite the fact that several pre-clinical trials incorporating miRNA therapies had been undertaken in the following years, out of those only a few miRNA therapeutics had progressed towards clinical development. Identifying the optimal miRNA candidate or the miRNA targets for every kind of illness is one of the most difficult issues in creating miRNA-based treatments. The present influx of data of genomes, proteomes, and transcriptomes in humans, and effective data analytics in integration through advanced soft-computing, artificial intelligence, and system biology technologies may assist in the discovery of critical miRNA targets for medicine preparation with precision. All of the growing corpus of information, along with the thorough pre-clinical evaluation helped via innovative delivery systems, would make miRNA therapies a natural premium as a clinical paradigm that will last for a longer period of time in precision oncology.

## Abbreviations

AGO3: argonaute RNA-induced silencing complex catalytic component 3
ASOs: antisense oligonucleotides
BCL2: B-cell lymphoma 2
CCND2: cyclin D2
DEmiRs: differentially expressed microRNAs
DOPC: 1,2-dioleoyl-sn-glycero-3-phosphatidylcholine
EGFR: epidermal growth factor receptor
GalNac: *N*-acetyl-galactosamine
HCC: hepatocellular carcinoma
HMED: human microRNA expression database
HOXD10: homeobox D10
LNA: locked nucleic acid
miRNA: microRNA
mRNA: messenger RNA
ncRNAs: non-coding RNAs
NGS: next generation sequencing
NLE: neutral-lipid emulsion
NSCLC: non-small cell lung cancer
OncomiRs: oncogenic miRNAs
pHLIP: pH low insertion peptide
pre-miRNAs: precursor microRNAs
RISC: RNA-induced silencing complex
TLRs: Toll-like receptors
UTR: untranslated region
VIRmiRNA: viral microRNA

## Declarations

### Copyright

© The Author(s) 2022.
